# Size-dependent retention of elongate mineral particles in human lungs: modeling and implications for risk assessment

**DOI:** 10.3389/fpubh.2025.1646016

**Published:** 2025-09-04

**Authors:** Andrey Korchevskiy, Richard Attanoos, Ann G. Wylie

**Affiliations:** ^1^Chemistry and Industrial Hygiene, Inc., Lakewood, CO, United States; ^2^Department of Cellular Pathology, University Hospital of Wales & School of Medicine, Cardiff University, Cardiff, Wales, United Kingdom; ^3^Department of Geology, University of Maryland, College Park, MD, United States

**Keywords:** asbestos, non-asbestiform particles, lung deposition, fiber length, fiber width

## Abstract

**Introduction:**

Lung deposition of elongate amphibole particles is an important process impacting the risk of cancer. There is, however, a significant gap in scientific literature characterizing the role of particle size in the differences observed for deposition and clearance rate in the human respiratory system. The purpose of the paper is to explore the relationship between size distribution of elongate mineral particles in human lungs compared to corresponding distribution in the airborne exposure.

**Materials and methods:**

Previously published information about lung deposition for amosite and crocidolite particles in various dimensional groups, collected by the team of Pooley and Clark was reanalyzed with application of recently developed methodologies for fiber size analysis. The new metric—deposition selection ratio (DSR) is proposed; it is found by dividing the size fraction of particles in lungs to the corresponding fraction in exposure. The DSR estimations were also compared to theoretical estimations of pulmonary deposition rates of particles based on the United States Environmental Protection Agency (US EPA) Multi-Path Particle Dosimetry (MPPD) model.

**Results:**

It was demonstrated that DSR values can be approximated by using log–log regressions with length and width of particles as independent variables. For non-asbestiform particles (cleavage fragments), the prediction of DSR from parametric and non-parametric models is demonstrated to be less than 1 (evidence of deselection in lungs). Negative correlation was found for DSR estimations and the theoretical predictions of pulmonary deposition rates by MPPD.

**Discussion:**

The observed data for size-specific lung deposition of elongate mineral particles can be used for quantitative estimates of risk and analysis of toxicokinetic processes in human lungs. The difference between theoretical model and observed fiber deposition pattern requires further adjustments in the methods to predict lung deposition of elongate particles.

## 1 Introduction

Mineral analysis of lung tissue is a well-established method for assessing prior dust exposures and it plays a central role in diagnosing and determining the causation of a wide variety of occupational and environmental diseases, including pneumoconiosis and cancers. Quantitative and qualitative measurements of retained asbestos fibers have been used to evaluate causation in mesothelioma and other asbestos-related diseases (1–7). Retained amphibole asbestos in the lungs has consistently been identified as a key biomarker of exposure and predictor of asbestos-related disease by retrospective exposure estimates (8–11).

The size and morphology of mineral particles are crucial in interpreting lung burden data (12), emphasizing the importance of detailed mineralogical analysis of elongate mineral particles in human lungs. However, the information on the size of fibers in human lung tissue remains limited. No original data in individual measurements of fiber sizes, as a rule, are available. The results of lung fiber size measurements are systematically reported as separate statistical values for length and width, but almost never in length/width matrices. In addition, a significant fraction of the lung burden size information reported is a result of SEM (scanning electron microscopy), and not TEM (transmission electron microscopy) analysis, which limits the full-size distribution characterization, because of a lack of visibility of very thin fibers with SEM.

In this context, the archive of a national referral center in the United Kingdom (UK), where concentrations of fibers in lungs have been measured by TEM since the 1970s, has a special value for the studies of lung size distribution. As a baseline for the analysis, in this paper we utilized size distributions of amosite and crocidolite in lungs reported by Fred Pooley, the founder of the fiber analysis program, and published by Pooley and Clark in 1980 (13).

In our analysis, we revisited data from Pooley and Clark applying advanced approaches to the fiber size analysis developed recently (12, 14–18). Pooley and Clark used airborne samples from South Arican crocidolite and amosite mines to compare with lung distribution. During the last few years, the dimensional database for various types of elongate mineral particles was created (18, 19). We augmented the analysis of Pooley and Clark by the inclusion of the dimensional database information for typical size distribution of amosite and crocidolite.

We suggest that the ratio of the size fraction of various types of elongate minerals in lung burden to that in the exposure can serve as an indicator of deposition/retention of particles in lungs, which can be preferential for some size groups, or deselecting for others. The meaning of “preferential” deposition is in the process of increasing of the fraction of specific size categories of particles in the lungs compared to exposure. Deselection is the process when size fraction for a specific category is decreasing. Preferential deposition and deselection have specific mechanisms. In particular, some particles have aerodynamic characteristics allowing them to penetrate deeper in the lungs and to be retained for longer periods. For some size fraction, aerodynamic and other characteristics would help the particles to be removed from the lungs immediately after breathing cycle, or to be cleared quickly. For the purpose of distinguishing between particles by their propensity to be deposited, we introduced a new parameter – a deposition selection ratio (DSR). This parameter is expected to be higher than 1 for preferential, and less than 1 for deselection processes in lungs. We determined the relationship between DSR and length and width of elongate particles. Pooley and Clark concluded that fibers of amosite and crocidolite in the lungs appeared to be “larger” and suggested this as a selective retention of longer fibers. Our closer analysis shows, however, that the higher preferences in the lung retention are given to longer, thinner particles (12). Short particles can also be deposited in lungs, but there is extensive literature highlighting that short fibers (< 5 microns) do not have dimensional or morphological characteristics to produce mesothelioma (20).

We paid special attention to the predicted deposition rate of asbestiform vs. non-asbestiform particles in lungs. Asbestiform fibers and non-asbestiform cleavage fragment mineral particles differ in their dimensional characteristics. The properties of amphibole asbestos minerals include a particular fibrous habit referred to as asbestiform. Asbestiform particles represent naturally occurring, polyfilamentous mineral growth habit in which fibers are formed of parallel fibrils in bundles that can be separated into smaller fibers and fibrils with hand pressure; asbestiform minerals are flexible in hand samples, and fibers have higher tensile strength than other habits of the same mineral. Non-asbestiform amphibole (cleavage fragments) are elongate mineral particles created by *fragmentation* that are bound in whole or in part by inherent planes of weakness. Cleavage fragments may be elongate but they do not have biological activity typical for natural asbestiform fibers and are not regulated as asbestos (21). The fragments also tend to be shorter and thicker on average than asbestos, have higher aerodynamic diameter, and higher correlation between length and width (emphasizing the mechanical shaping processes vs. natural growth). Cleavage fragments occur in small quantities with asbestiform fibers in most asbestos mine products.

The objective of this study is to explore the relationship between the size distribution of elongate mineral particles in human lungs compared to corresponding distribution in the airborne exposure.

We hypothesize that cleavage fragments are typically deselected from lung deposition, which would explain that the elongate particle populations in lungs have characteristics of asbestiform particles (12). In this paper, we will use both parametric and non-parametric methods to test our hypothesis.

We compared our estimations of DSR values with the pulmonary deposition rate of fibers calculated by the US EPA MPPD software. The MPPD model is theoretical and based on perceived scientific knowledge on the processes governing the deposition of fibers in lungs (22). However, our hypothesis is that theoretical models of particle transport cannot fully reflect the size-specific distribution of EMPs in lungs. The observed DSR can be a better practical tool for exploration and prediction of fiber transport and retention than existing quantitative models.

## 2 Materials and methods

In 1976, a collaborative case–control analysis was conducted between multiple United Kingdom pathologists, with the UK Mesothelioma Panel, the Medical Research Council Pneumoconiosis Unit, and University College, Cardiff. Multiple samples from mesotheliomas and wet lung tissue were collected for analysis (optimally three lung regions—lung upper lobe, apex lower lobe, and lung base), or a 2 cm whole sagittal lung slice (23, 24). Lung tissue for analysis was collected for controls that were comprised of subjects who had died of cerebrovascular disease, or lung cancer (56 control cases). The UK mesothelioma panel pathologists verified 93 submitted cases as mesothelioma with 86 having adequate lung tissue for mineral analysis. Of the confirmed mesotheliomas, 82% were men with a mean age at death of 62 years. The anatomic site was known in 60 cases: 90% were pleural with a male:female ratio of 4:1, and 10% peritoneal site with male:female ratio of 1:1. 74% had an occupational or known positive exposure history, 3% no exposure history, and 23% unknown exposure details.

The lung burden samples were obtained by Pooley and Clark from mesothelioma cases and controls referred to the Cardiff pneumoconiosis unit. The methodology of sample preparation and analysis has been preserved in the Cardiff laboratory since the 1970s. Fiber analysis was performed on formalin-fixed wet lung tissue specimens. Mineral analysis was conducted on ‘pooled’ lung samples, characterizing the overall content of fibers in the lungs vs. specific parts of the lungs.

Formalin-fixed wet tissue was directly digested in potassium hydroxide until organic tissue was removed. Following digestion, the sample was washed with distilled water and centrifuged; the process was repeated at least three times, with sequential removal of the supernatant and replacement with distilled water. The filter residue sample was prepared for examination on the electron microscope by transfer to a nucleopore membrane, filtered, then carbon coated ready for sections to be taken on TEM grids.

Samples were then examined with a Phillips 301 transmission electron microscope (TEM) equipped with an energy dispersive x-ray analyzer (EM/EDXA). A low power evaluation of the grid was performed to ensure sample uniformity. Elongate structures with parallel sides, an aspect ratio of at least 3:1, and > 0.5 μm in length were measured in two dimensions at a magnification of 20,000x and recorded. Only amosite and crocidolite data from the Pooley and Clark analysis were used for this paper, because full dimensional characteristics of the fibers were available.

The data published by Pooley and Clark (13) were used to reconstruct fiber-by-fiber datasets for amosite and crocidolite particles found in human lung tissue, as well as in airborne samples from Pomfret mine (South Africa, crocidolite), and Penge mine (South Africa, amosite). The summarized data from Pooley, Clark (13) is available in Supplementary Table S1. A total of 2,000 particles was generated for each of the mineral types and source (two sets for amosite, two sets for crocidolite). Monte Carlo simulation was used to generate combinations of length and width for particles corresponding to the length/width matrix of size distribution published by Pooley and Clark.

As described above, we assume that the data included 86 mesothelioma cases and 56 controls. Following Pooley and Clark (13) we assume that dimensional distribution of particles in the lungs of combined cases and controls for amosite and crocidolite reflected the process of exposure, deposition, and clearance of particles that can be compared to dimensional distribution of corresponding types of mineral particles in environmental samples.

The dimensional distribution of lung burden was compared to airborne data reported by Pooley and Clark, along with the airborne and bulk TEM samples from the dimensional database described elsewhere (referred to below as “dimensional database”) (16–18). For dimensional characterization, 32 size categories were used: for length, the categories of <1, 1–2, 2–3, 3–4, 4–6, 6–8, 8–10, >10 μm, and for width, <0.125, 0.125–0.250, 0.250–0.375, and >0.375 μm.

Monte Carlo simulation was used for reconstruction of individual datapoints (software in Python). A total of 2000 datapoints were generated for each mineral type and media (airborne vs. lung concentrations). The distributions of length and width in each of 32 size groups were assumed to be log-normal.

The US EPA model was tested for each size category to find a predicted pulmonary deposition rate of particles separately for amosite and crocidolite. For this purpose, the data from the dimensional database for amosite and crocidolite were used to calculate count median diameter and median aspect ratio for each of the 32 size categories. Version 3.04 of the software was used. Deposition was calculated by using the Yeh/Schum Symmetric model (25), assuming an FRC (Functional Residual Capacity) of 3,300 mL, and a URT (Upper Respiratory Tract) volume of 50 mL. We also assume an upright posture with a breathing frequency of 12 per minute, a standard tidal volume of 625 mL, and a nasal breathing scenario. The pulmonary deposition fraction is defined as the fraction of inhaled aerosol mass (or fraction of the number of inhaled particles, given a monodisperse distribution) that is deposited in the pulmonary region of the respiratory tract.

The sources of data (from the dimensional database) included for amosite and crocidolite are provided in Appendix A (Table 1A).

The fraction of each of 32 size groups for amosite and crocidolite was calculated from lung burden data from Pooley and Clark, airborne data from Pooley and Clark, and data from dimensional database.

We introduced a parameter that we called deposition selection ratio (DSR) that was calculated for each size group following Equation 1:


(1) $${\mathrm{DSR}}_{i}=f_{i}(\text{lungs})/f_{i}(\text{exposure})$$

Where i—size group,

f_i_(lungs)—fraction of elongate particles of the group i in lungs, and

f_i_(exposure)—fraction of elongate particles of the group i in the exposure.

The following regression model was proposed for the evaluation of the role of length and width in the selective deposition of EMPs with various size characteristics:


(2) $$\log_{10}(\mathrm{DSR}+0.001)=A+{\text{Blog}}_{10}(\text{length})+{\text{Clog}}_{10}(\text{width})$$

The use of log-transformation for DSR, length, and width in our analysis is supported by the log-normal distribution of these parameters. The log-normal distribution for length and width of asbestos fibers was, in particular demonstrated by Cheng in 1986 (26). We also confirmed that parameter DSR is distributed log-normally (Kolmogorov–Smirnov test with *p* = 0.14).

Assuming that a good fit of Equation 2 would provide an approximation of the selective size-dependent deposition of particles in lungs, we used this equation for all particles included in the dimensional database. Then we explored the difference in deposition rate between various habits of particles and the two sources of dimensional data.

Aerodynamic diameter of particles was calculated based on Timbrell (27) model (Equation 3):


(3) AD=66W(AR/(2+4AR))2.2x(ρρ/ρ0)0.5

where AD = aerodynamic diameter, W = measured width of the EMP, AR = length/width, ρρ = density in g/cm^3^, and ρ0 = 1.0 g/cm^3^.

We used two categories of elongate mineral particles for comparison: “criteria” particles that corresponds to the value of Equation 4:


(4) $$2.99\log_{10}(\text{length})-5.82\log_{10}(\text{width})-3.80>=0$$

and “non-criteria” particles for all other EMPs. Criteria particles serve as a close approximation of the “asbestiform” particle set excluding the possible data noise (15).

Pearson index for particle samples was determined as a correlation coefficient in Equation 5:


(5) logW=FlogL+C

Where W is the width, L is the length of the particles (assumed particles longer than 2 μm, with width ≥0.05 μm, width ≤3 μm, length/width ≥3) (15, 17).

Let us assume that CriteriaFraction variable is the fraction of criteria particles in a sample, and that the PearsonIndex is the Pearson index of the sample, calculated as above. In this case, we can use the following decision rule to determine a habit for the sample:

If CriteriaFraction≥0.58PearsonIndex + 0.12, then the habit is **asbestiform**.

If PearsonIndex ≥ 0.3, PearsonIndex *≤* 0.5, and CriteriaFraction ≥ 0.2 and CriteriaFraction *≤* 0.3, or if PearsonIndex ≥ 0.4, PearsonIndex *≤* 0.5, and CriteriaFraction ≥ 0.1 and CriteriaFraction *≤* 0.2, the habit is **undetermined**.

Otherwise, the habit is **non-asbestiform** (17).

We used two types of classification methods for asbestiform and non-asbestiform habits. For individual particles, we used subdivision of particles as criteria vs. non-criteria as approximation for asbestiform vs. non-asbestiform particles classification.

For the samples, we used combined methods with criteria fraction and Pearson index as indicated above.

We also calculated average DSR values for various mineral particles. The values for each mineral type were then compared with reported biopersistence (28) and mesothelioma potency (29).

The data for mesothelioma potency and biopersistence of fibers are provided in Table 1.

**Table 1:** Mesothelioma potency factor R_M_ (%) and biopersistence (years) of different mineral particles under acellular dissolution conditions in Gamble’s solution.

Mineral type	R_M_ (%)	Biopersistence (years)	References
Crocidolite (South Africa and Australia)	0.52*	66***	*Darnton, 2023 (29) **Korchevskiy et al., 2019 (37) ***Gualtieri, 2018 (28) ****Korchevskiy and Wylie, 2023 (38)
Amosite (South Africa)	0.11*	77***	
Chrysotile (Quebec)	0.0009*	0.3***	
Libby amphiboles (Libby, Montana)	0.03 *	49 (as for asbestiform tremolite)***	
Anthophyllite asbestos (Russia and Finland)	0.056**	245***	
Erionite (Karain)	4.67**	181***	
Balangeroite (Italy)	0.045****	55****	

We compared DSR values with corresponding estimations of pulmonary deposition rate based on the MPPD model and drew conclusions about the validity of MPPD for a prediction of the probability of elongate mineral particles deposition in lungs.

## 3 Results

In this section, we provide the results of our study. In particular, we determined the DSR parameter reflecting the relationship between size fraction of elongate particles in the lungs vs. typical airborne distribution. We demonstrated consistency of the DSR estimations by comparing two sources of dimensional information for airborne fibers (original data from Pooley and Clark, and the dimensional database). Using the dimensional data, we compared the morphological habit of particles in the lungs and in the airborne exposure. We modeled DSR as a function of length and width and demonstrated that this parameter tends to increase with length and decrease with width. We also estimated DSR parameters for particles with different habit: asbestiform vs. non-asbestiform. We showed that our estimate of DSR for particles with different habit can be performed non-parametrically with results comparable to parametric values. We showed that combined DSR (initially derived for crocidolite and amosite) can play as an independent variable in the regression equation predicting mesothelioma potency for various mineral types of fibers. We also tested the relationship between observed DSR values and pulmonary deposition rate calculated by US EPA MPPD model.

### 3.1 Calculation of the ratios between lung and exposure size-specific frequencies

Fractions f_i_ for various size groups, mineral types of fibers, sources of information, and media (airborne, airborne and bulk, lung burden), along with the ratios (DSR values) are given in Table 2.

**Table 2:** Fractions f_i_ for various size groups, mineral types of fibers, sources of information, and media (airborne, airborne and bulk, lung burden), along with the ratios (DSR values; particles with length/width ≥3, width ≥0.05 μm, width ≤3 μm).

Length (μm)	Width (μm)	Frequency in lungs (%)	Frequency in airborne samples, Pooley (13) (%)	Frequency in dimensional database (airborne and bulk samples, by TEM; %)	Ratio lungs to airborne (DSR)	Ratio lungs to dimensional database (DSR)
Crocidolite						
<1	<0.125	11.91	18.91	17.80	0.63	0.67
1–2		26.07	21.70	8.17	1.20	3.19
2–3		14.04	5.28	2.74	2.66	5.12
3–4		8.84	2.48	2.17	3.57	4.08
4–6		6.93	2.78	2.41	2.49	2.88
6–8		1.61	0.83	1.78	1.95	0.91
8–10		0.76	0.50	0.87	1.53	0.88
>10		0.57	0.00	2.15	N/A	0.27
<1	0.125–0.250	1.09	4.99	7.63	0.22	0.14
1–2		4.50	14.14	4.82	0.32	0.93
2–3		4.74	7.43	3.10	0.64	1.53
3–4		3.66	4.63	2.40	0.79	1.52
4–6		4.49	3.75	3.90	1.20	1.15
6–8		2.25	1.49	4.08	1.52	0.55
8–10		1.21	0.67	1.91	1.82	0.63
>10		1.12	0.49	4.87	2.30	0.23
<1	0.250–0.375	0.00	0.17	0.20	0.00	0.00
1–2		0.43	1.81	1.01	0.24	0.43
2–3		0.64	1.32	0.90	0.49	0.72
3–4		0.65	0.66	1.25	0.98	0.52
4–6		0.96	0.98	1.07	0.99	0.90
6–8		0.71	0.33	4.80	2.15	0.15
8–10		0.64	0.67	1.53	0.95	0.42
>10		0.94	0.49	3.46	1.92	0.27
<1	>0.375	0.00	0.00	0.00	N/A	N/A
1–2		0.00	0.15	0.11	0.00	0.00
2–3		0.06	0.99	0.49	0.06	0.12
3–4		0.07	0.83	0.69	0.08	0.10
4–6		0.13	0.49	2.64	0.26	0.05
6–8		0.07	0.66	3.57	0.10	0.02
8–10		0.19	0.17	2.05	1.15	0.09
>10		0.38	0.33	5.45	1.15	0.07
Amosite						
<1	<0.125	2.24	6.86	6.74	0.33	0.33
1–2		8.30	5.58	3.61	1.49	2.30
2–3		5.53	2.71	0.92	2.04	6.00
3–4		3.71	0.96	0.75	3.87	4.94
4–6		2.64	1.11	0.44	2.38	6.05
6–8		0.81	0.34	0.22	2.40	3.70
8–10		0.41	0.32	0.05	1.26	8.35
>10		0.21	0.81	0.19	0.26	1.08
<1	0.125–0.250	1.09	4.80	7.37	0.23	0.15
1–2		8.84	12.13	7.71	0.73	1.15
2–3		9.22	5.73	2.59	1.61	3.55
3–4		7.92	3.85	2.23	2.06	3.55
4–6		6.15	4.14	3.27	1.49	1.88
6–8		2.97	2.01	1.87	1.47	1.59
8–10		2.10	1.44	0.70	1.46	2.98
>10		1.01	1.45	2.45	0.70	0.41
<1	0.250–0.375	0.27	0.64	0.75	0.42	0.36
1–2		2.43	5.76	4.61	0.42	0.53
2–3		4.18	4.31	2.50	0.97	1.67
3–4		3.12	2.73	1.41	1.14	2.22
4–6		4.73	3.51	0.97	1.35	4.87
6–8		3.24	0.50	3.27	6.46	0.99
8–10		1.29	0.96	0.78	1.34	1.66
>10		2.47	1.13	3.56	2.18	0.69
<1	>0.375	0.00	0.00			
1–2		0.34	2.70	1.53	0.13	0.22
2–3		1.49	6.05	3.66	0.25	0.41
3–4		1.75	3.37	2.52	0.52	0.69
4–6		3.17	4.93	9.12	0.64	0.35
6–8		1.49	2.85	7.83	0.52	0.19
8–10		1.63	2.08	3.20	0.78	0.51
>10		5.42	4.52	13.19	1.20	0.41

Table 2 allows analysis of various estimations of the DSR parameter that we calculated for various size groups of amosite and crocidolite, with various assumptions. Further we will demonstrate the relationship that exists between DSR values and length and width of elongate particles.

### 3.2 Correlation between different estimates of DSR values

In order to assess overall consistency of the fiber retention properties, we determined correlations between various estimates of DSR. The correlations are shown in Table 3.

**Table 3:** The linear correlations between log-transformed DSR values determined for various mineral types and sources of information.

Mineral type	Parameter	Crocidolite		Amosite	
		Pooley (13) lung to airborne ratio	Lung to dimensional database ratio	Pooley (13) lung to airborne ratio	Lung to dimensional database ratio
Crocidolite	Pooley, Clark lung to airborne ratio	1	0.84	0.75	0.58
	Lung to dimensional database ratio	0.84	1	0.63	0.76
Amosite	Pooley, Clark lung to airborne ratio	0.75	0.63	1	0.73
	Lung to dimensional database ratio	0.58	0.76	0.73	1

All correlations in Table 3 are statistically significant at *p* < 0.05. It can be seen that relationships between various estimations of DSR values are generally strong; in particular, the correlation between DSRs for crocidolite estimated from original Pooley and Clark data, and from the full dimensional database are both 0.84 (R^2^ = 0.70). This correlation is slightly lower for amosite (R = 0.73) but also equivalent for both sources of data. Correlation between ratios for amosite and crocidolite in the Pooley and Clark data is 0.75, and 0.76 for the dimensional database information.

### 3.3 Determination of a habit of particles in airborne samples and lungs

Table 4 contains values of criteria particle fraction, Pearson index, and decision rule on the habit for airborne and lung concentrations reported by Pooley and Clark (13). As was indicated in the Materials and Methods section, the particles were determined to be asbestiform if CriteriaFraction-(0.58xPearsonIndex + 0.12) exceeds zero.

**Table 4:** Determination of a morphological habit for different datasets on elongate mineral particles.

Mineral type	Media	Criteria fraction	Pearson Index	Decision rule and habit determination
Amosite	Airborne	0.61	0.30	>0 (asbestiform)
	Dimensional database	0.68	0.31	>0 (asbestiform)
	Lung	0.73	0.47	>0 (asbestiform)
Crocidolite	Airborne	0.88	0.23	>0 (asbestiform)
	Dimensional database	0.91	0.30	>0 (asbestiform)
	Lung	0.96	0.35	>0 (asbestiform)

We can see that all populations in the Pooley and Clark analysis are asbestiform. Criteria fraction of particles in lungs is high (73–96%), and there is also a trend of increase of this fraction in lungs compared to both airborne and dimensional database samples which include TEM measurements of both bulk and airborne samples of amosite or crocidolite. The decision rule based on both Pearson index and Criteria fraction is positive for asbestiform habit for all airborne and lung samples. It is noteworthy, though, that the correlation between length and width for asbestiform particles seems to be higher in lungs than in airborne samples. This can be explained by the process whereby asbestiform particles in lungs are filtered by physical processes evident when additional relationships between different dimensions of particles are introduced. We can see it as two processes impacting the deposition of elongate mineral particles in lungs:

(1) Non-asbestiform particles are not deposited or are quickly removed (along with non-elongate structures), and
(2) Asbestiform particles are selected by their dimensions for transfer to the lung and deposition, and the relationship between length and width is slightly strengthened with deposition.

### 3.4 DSR values as a function of length and width of particles

Combining two values of the ratio as independent estimate of true DSR level, and combining amosite and crocidolite, we can establish the following regression Equation 6:


(6) $$\begin{array}{l} \log_{10}(\mathrm{DSR}+0.001)=-1.09+0.53\log_{10}(\text{length})-\\ 1.03\log_{10}(\text{width}) \end{array}$$

(R = 0.62, R^2^ = 0.38, *p* < 0.000001).

While the correlation is of medium level, it is reasonable to suggest that Equation 6 reflects the central tendency of the observed process; the uncertainty is caused by the fact that exposure characteristics can be measured only approximately.

**Figure 1:**
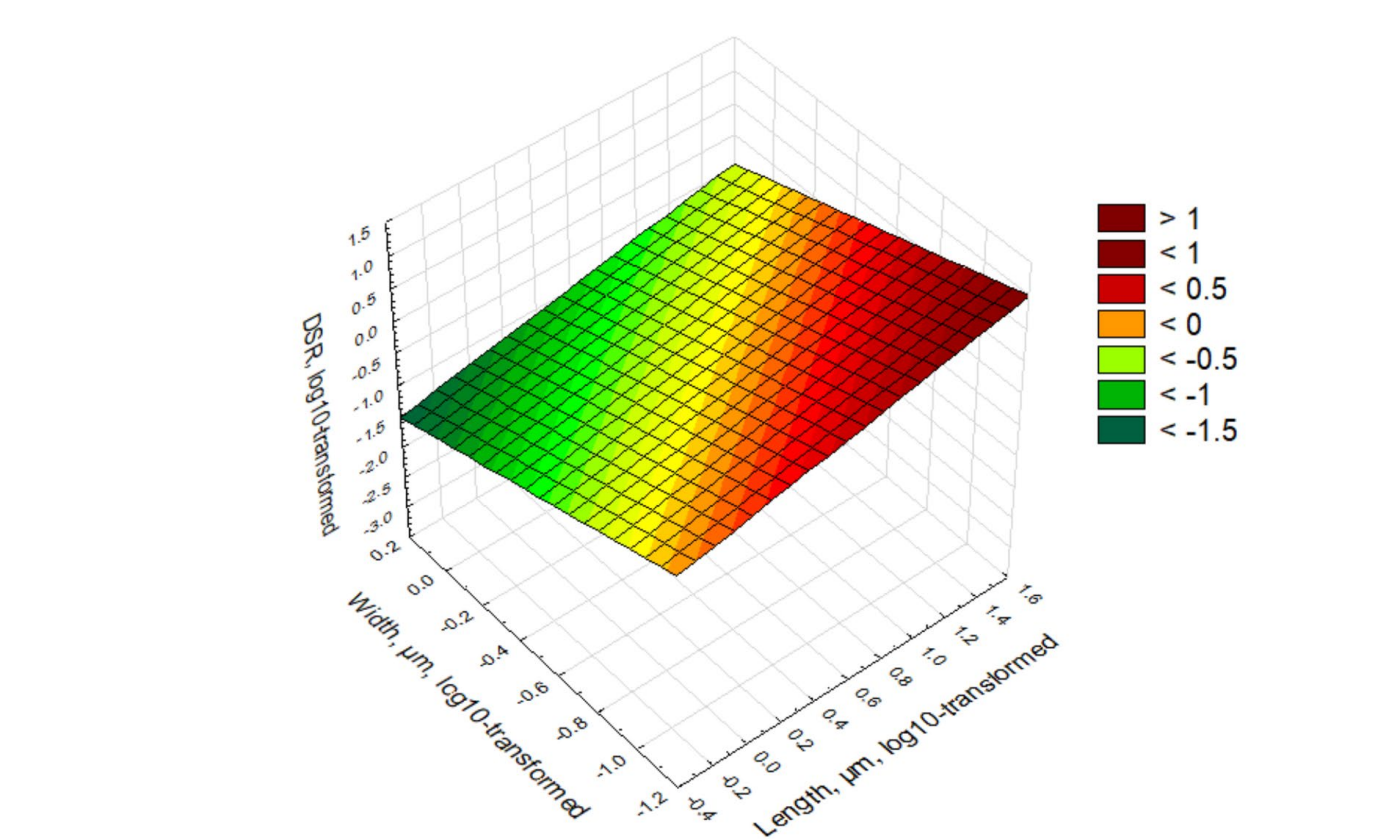
Relationship between length, width, and DSR values. DSR ranges are given in the legend.

The relationship is illustrated in Figure 1.

Figure 1 illustrates the relationship between log-transformed DSR values from log-transformed length and width of elongate particles. The slope of the linear plane shows that DSR increases with length and decreases with width (as it is reflected in the green color at high width, low length, and red color for low width, high length). The lowest DSR corresponds to length of 0.4–0.5 μm and width of 1.5 μm. Short and thick particles as a rule will not be deposited in the lungs and their fraction will decrease compared to the airborne exposure. The highest DSR will be at length higher than 40 μm and width less than 0.1 μm. Long, thin particles, based on the model, have the highest propensity to be deposited in lungs, and their fraction will increase.

Separately for crocidolite and amosite, the following regression equations can be developed (based on the data from Table 2):

#### 3.4.1 Crocidolite


(7) $$\begin{array}{l} \log_{10}(\mathrm{DSR}+0.001)=-1.67+0.76\log_{10}(\text{length})-\\ 1.45\log_{10}(\text{width}) \end{array}$$

(R = 0.72, R^2^ = 0.51, *p* < 0.00001).

#### 3.4.2 Amosite


(8) $$\begin{array}{l} \log_{10}(\mathrm{DSR}+0.001)=-0.52+0.30\log_{10}(\text{length})-\\ 0.63\log_{10}(\text{width}) \end{array}$$

(R = 0.61, R^2^ = 0.37, *p* < 0.00001).

It is remarkable that the ratio between coefficients by width and length in Equations 7, 8 are close (1.9 vs. 2.1, respectively). This correspondence between ratios of coefficients shows that there is a commonality in the deposition process for crocidolite and amosite (though specific coefficients are different).

All correlation coefficients in Equations 6–8 are statistically significant. Based on Akoglu (30), the correlation coefficients in these equations can be ranked as moderate (or at least “fair”) to strong or even very strong by several scales used in scientific literature. However, the coefficients of determination between 37% (as for Equation 8) to 51% (as for Equation 7) are not very high. Below we will use both parametric and non-parametric estimations of the relationships between DSR, length, and width, to check our overall conclusions.

##### 3.5 Estimation of DSR values for elongate particles with different morphology

We applied Equation 6 to all amphibole particles in the dimensional database.

**Figure 2:**
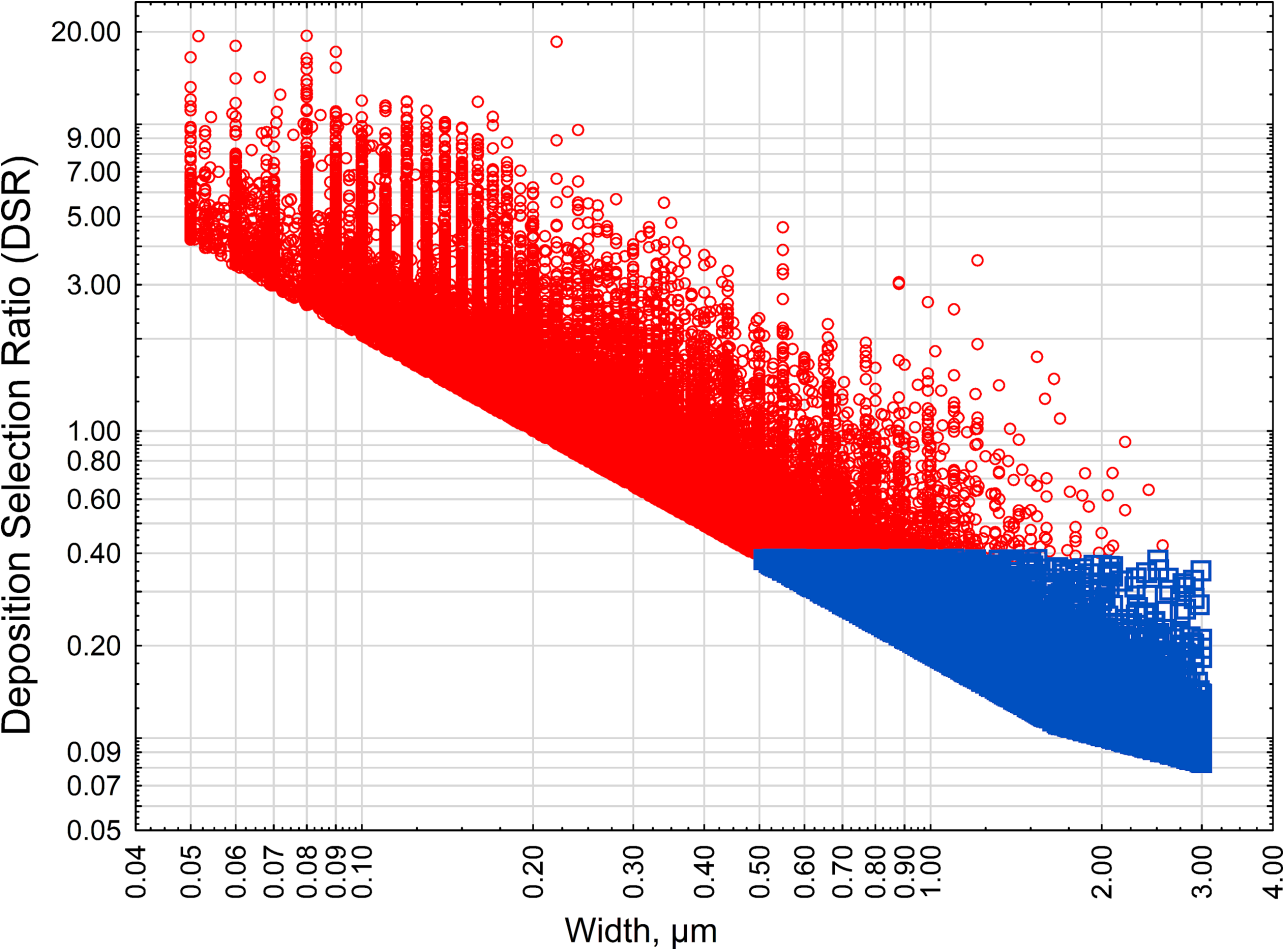
DSR for various habits of amphibole particles. Red squares—criteria particles, blue circles—non-criteria particles.

Figure 2 demonstrates the distribution of all particles longer than 5 μm by their width and modeled DSR value. The particles are subdivided by “criteria” and “non-criteria” particles categories (approximating classification as asbestiform and non-asbestiform particles).

As we can see, 100% of non-criteria particle have DSR < 0.5 (deselection), and criteria particles have mixed pattern of selection, with 50% having DSR > 1 (preferential selection). Because particles, and not samples, were plotted in Figure 2, no “mixed” or “undetermined” categories were considered for the classification.

Figure 2 shows that amphibole particles with a morphology typical for the non-asbestiform (or cleavage fragments) variety will have a lower propensity to be deposited in human lungs. Red circles indicate particles that would be estimated as non-asbestiform based on the methodology by Wylie et al. (31). Blue squares are particles with typical non-asbestiform dimensions (short, thick particles). Figure 2 demonstrates relationship between DSR for both types of particles. Deselection of cleavage fragments from lung deposition is caused by dimensional differences. As we demonstrated previously, long and thin particles will have higher probability for deposition in the pulmonary area. The biological meaning of this process can be seen in aerodynamic characteristics. The aerodynamic diameter of elongate particles changes inversely with a square root of the aspect ratio, making elongate particles significantly more mobile in human lungs (27). Thin particles appear to have better penetration potential in a complex topology of human lungs (12). Long particles, at the same time, are more prone to be deposited when in contact with surfaces, and they are in many cases not cleared from the lungs efficiently.

##### 3.6 DSR values as one of possible predictors for mesothelioma potency

Table 5 contains average DSR values for major mineral types of EMPs.

**Table 5:** Average DSR values.

Mineral type	Average DSR (Standard error)
Crocidolite	1.78 (0.008)
Amosite	1.36 (0.009)
Chrysotile	1.92 (0.014)
Libby amphiboles	0.65 (0.02)
Anthophyllite asbestos	0.62 (0.02)
Erionite (Karain)	1.94 (0.02)
Balangeroite	0.69 (0.03)

Based on this calculation, we can demonstrate that by using DSR, we can model mesothelioma potency R_M_ value, if biopersistence from Table 1 were also used.

The following regression equation (Equation 9) can be proposed:


(9) $$\log_{10}(R_{M})=-3.07+3.01\log_{10}(\mathrm{DSR})+1.13\log_{10}(\text{Bioper})$$

Where


Bioper–biopersistence(years)

(R = 0.96, R^2^ = 0.93, *p* < 0.004).

**Figure 3:**
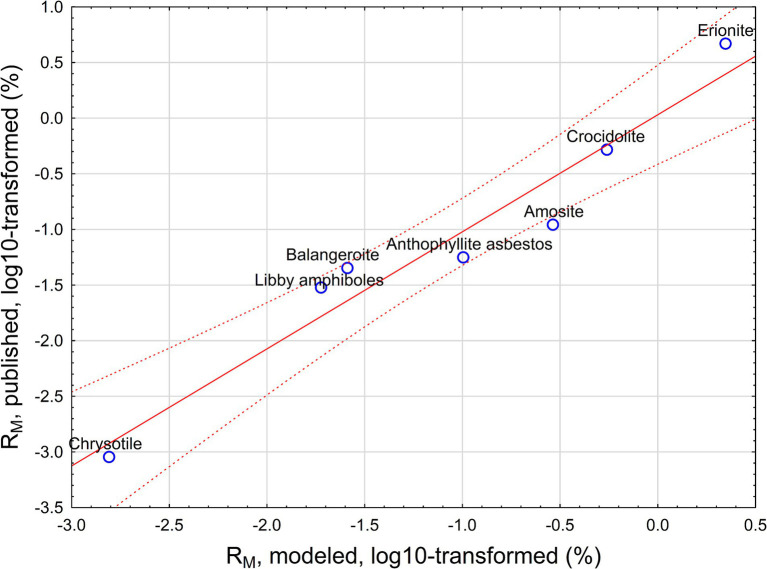
Predicted (from Equation 9) and published mesothelioma potency for various mineral fibers.

The relationship between predicted and observed potency factors based on Equation 9 is provided in Figure 3.

Our analysis proves that the DSR value has toxicological meaning, providing a possible relationship between deposition rate of EMPs, their biopersistence, and mesothelioma risk (potency). DSR is a value estimated for crocidolite and amosite in this paper. However, we can assess how Equation 6 can be interpreted for various mineral types of fibers, including chrysotile, anthophyllite, balangeroite, and erionite. We demonstrated that we can model mesothelioma potency R_M_ as a function of DSR and biopersistence of fibers. Strong correlation between predicted and observed R_M_ suggest that selective deposition of particles with various sizes in the lungs (reflected by DSR) can serve as an explanation of the role of length and width in differences of mesothelioma potency between various mineral types of fibers.

###### 3.7 Non-parametric estimation of DSR values for asbestiform and non-asbestiform particles

Because the correlation coefficient in regression Equation 6 is statistically significant, but not remarkably high, we attempted to use a non-parametric estimates of the relationship between length, width, and DSR values. In particular, we utilized direct estimations of ratios for crocidolite found between Pooley and Clark (13) lung and airborne concentrations to be projected on the dimensional database. For each crocidolite or non-asbestiform riebeckite in the dimensional database, we calculated DSR according to the ratios from Table 2 (column 6, crocidolite section of the table).

**Figure 4:**
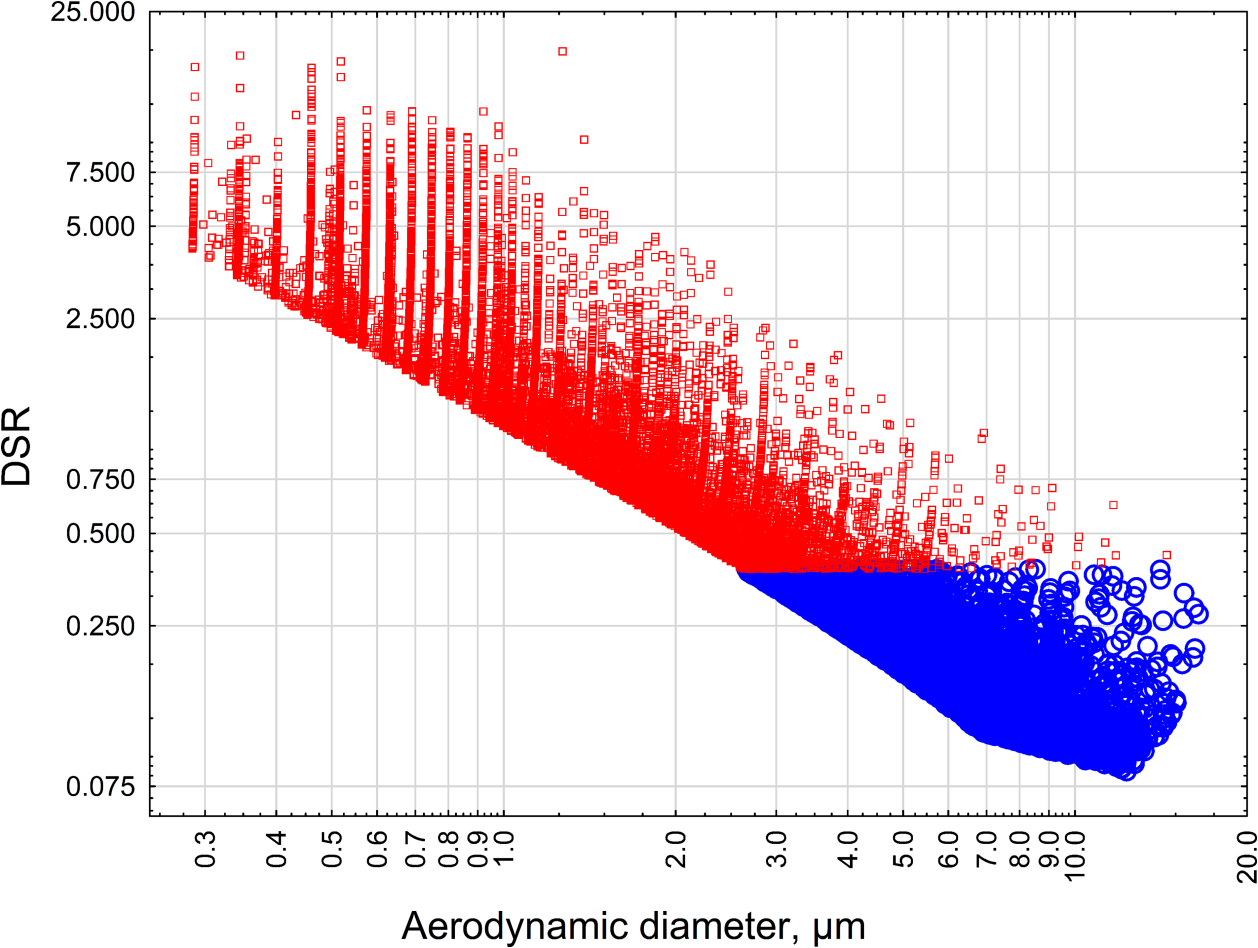
The relationship between the aerodynamic diameter of particles and estimated DSR for criteria and non-criteria particles longer than 5 μm, with width ≥0.05 μm, width ≤3 μm, length/width ≥3:1, for crocidolite and riebeckite. Red squares—crocidolite particles, blue circles—non-asbestiform riebeckite particles.

Figure 4 demonstrates the relationship between aerodynamic diameter and estimated DSR for criteria and non-criteria particles longer than 5 μm, with width ≥0.05 μm, width≤3 μm, length/width≥3:1, for crocidolite and riebeckite.

The analysis confirms the results of previous modeling. All non-asbestiform particles have DSR < 0.5, reflecting their deselection from lungs. To the contrary, the majority of asbestiform particles have DSR > 0.5, and about half of them exceed 1 (preferential selection).

Also, as can be seen from Figure 4, the majority of non-asbestiform particles in our analysis have aerodynamic diameter > 4 μm, above the respirability cutpoint, and the majority of asbestiform particles are respirable, to the contrary (32).

This way we confirmed that our conclusion about non-asbestiform particles having the propensity to be deselected during the lung deposition process would stand even if we use non-parametric methods for determining relationships between length, width, and DSR. We also confirmed the role of aerodynamic diameter in toxicological reasoning of the differences between asbestiform and non-asbestiform elongate particles.

#### 3.8 Comparison between DSR values and pulmonary deposition rate by US EPA MPPD

We compared DSR values with the US EPA MPPD estimates of pulmonary deposition rate for specific size groups of particles.

The MPPD values for deposition rate in amosite and crocidolite, according to particle size distribution are provided in Table 6.

**Table 6:** Deposition rate of EMPs according to the US EPA MPPD.

Length (μm)	Width (μm)	Crocidolite			Amosite		
		Geometric mean width (μm; GSD)	Average aspect ratio	Pulmonary deposition rate	Geometric mean width (μm; GSD)	Average aspect ratio	Amosite pulmonary deposition rate
<1	<0.125	0.07	9.33	0.11	0.09	7.03	0.09
1–2		0.07	18.03	0.09	0.09	16.84	0.07
2–3		0.08	33.86	0.07	0.09	28.66	0.05
3–4		0.08	46.14	0.06	0.09	38.44	0.05
4–6		0.08	61.33	0.05	0.10	54.89	0.05
6–8		0.09	79.81	0.03	0.11	63.90	0.05
8–10		0.09	101.61	0.02	0.11	83.78	0.05
>10		0.09	305.53	0.01	0.09	576.30	0.08
<1	0.125–0.250	0.15	4.84	0.08	0.16	4.47	0.08
1–2		0.15	8.77	0.07	0.18	8.11	0.07
2–3		0.15	16.31	0.06	0.18	13.64	0.06
3–4		0.16	21.41	0.06	0.17	20.80	0.06
4–6		0.17	29.84	0.06	0.19	26.77	0.06
6–8		0.17	39.30	0.06	0.21	32.92	0.06
8–10		0.18	50.59	0.06	0.20	45.53	0.06
>10		0.17	185.11	0.06	0.19	88.43	0.06
<1	0.250–0.375	0.26	3.36	N/A	0.26	3.42	0.08
1–2		0.29	4.98	0.07	0.30	4.91	0.07
2–3		0.31	8.13	0.07	0.30	8.22	0.07
3–4		0.31	11.41	0.07	0.32	10.86	0.07
4–6		0.30	17.05	0.07	0.30	17.16	0.07
6–8		0.30	23.15	0.07	0.29	23.01	0.07
8–10		0.30	29.87	0.07	0.30	29.97	0.07
>10		0.30	80.04	0.07	0.31	54.38	0.07
<1	>0.375						
1–2		0.41	3.87	0.09	0.47	3.57	0.09
2–3		0.45	5.72	0.10	0.54	4.75	0.10
3–4		0.49	7.25	0.11	0.64	5.62	0.12
4–6		0.55	10.00	0.10	0.62	8.86	0.11
6–8		0.58	12.44	0.10	0.63	11.07	0.11
8–10		0.59	15.94	0.10	0.67	14.06	0.09
>10		0.69	34.25	0.07	0.68	35.30	0.06

**Figure 5:**
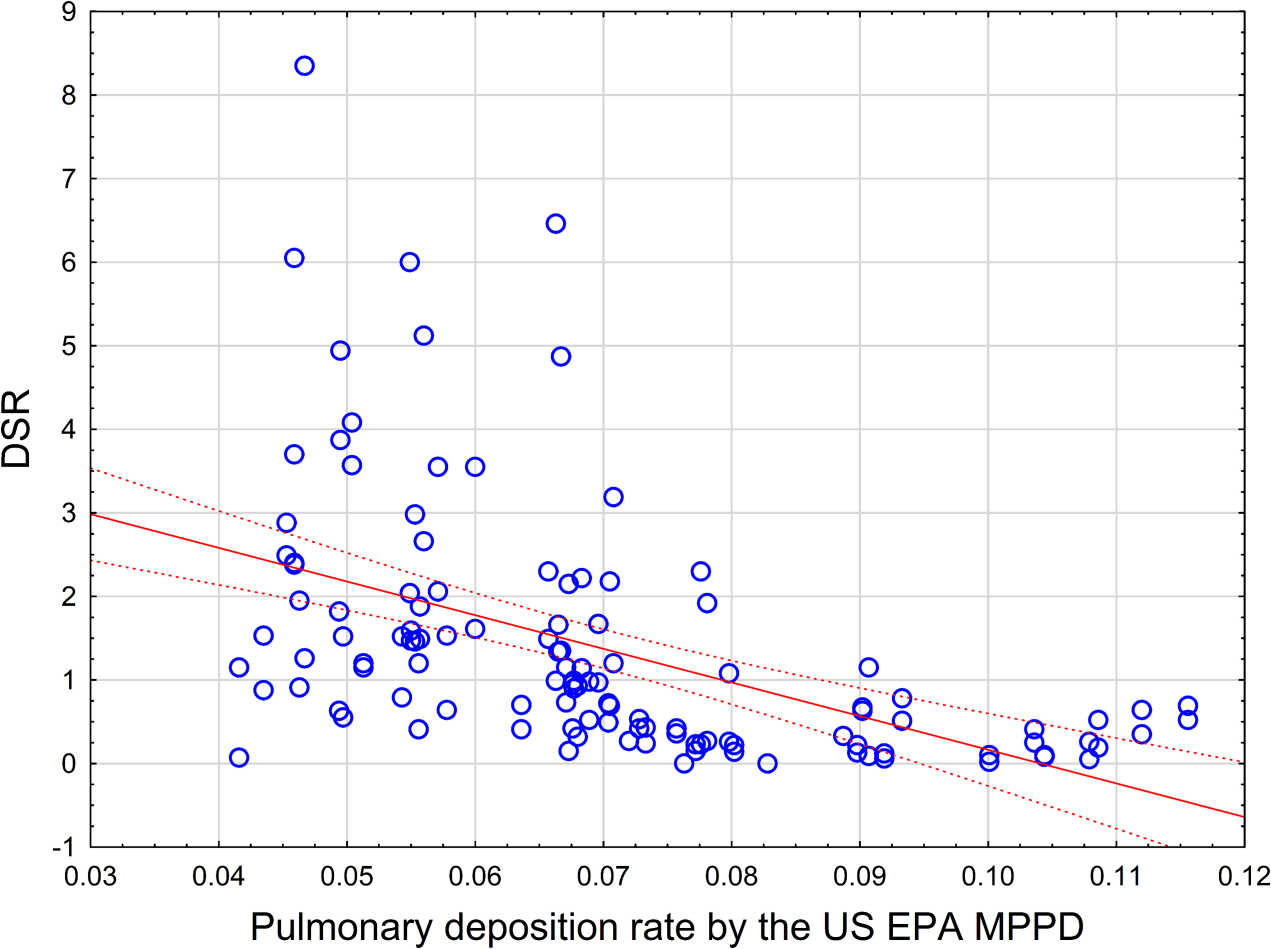
Pulmonary deposition rate by the US EPA MPPD in comparison with observed DSR values for 32 length/width groups (represented by blue circles). Red line—linear regression equation, dotted line 95% CI.

The relationship between our estimations of DSR in comparison to the US EPA MPPD pulmonary deposition rate value are shown in Figure 5. For each size group and mineral type (amosite and crocidolite), one value of US MPPD pulmonary deposition rate and two values of DSR (two right columns from Table 2) were utilized for the regression analysis.

As we can see, there is no meaningful statistical relationship between theoretical (MPPD) and observed size-specific selection values. The relationship between MPPD and DSR values is actually negative, with R = −0.5, R^2^ = 0.25, *p* < 0.0000001. MPPD also negatively, though weakly, correlates with frequencies of fiber sizes in lungs by Pooley and Clark: R = −0.23, R^2^ = 0.05, *p* < 0.01.

Theoretically, we expect that the MPPD model would predict the ratio between frequency of specific size ranges of length and width of elongate mineral particles, as related to the exposure size distribution. On the contrary, the theoretical MPPD model predicts a reversed order of frequencies.

## 4 Discussion

The inhomogeneity of inhaled aerosolized EMPs means that the particles have differing potentials for penetrating the various regions of the respiratory tract, including the tracheobronchial site, alveolar region, lung interstitium, pleural tissue, and other organs in the body. The likelihood of elongate mineral particles to be found in lung burden samples depends on the prior exposures and their physical and chemical properties. It is essential to differentiate carcinogenic EMP from other mineral dusts based on the properties that govern an EMP’s ability to reach target tissues (along with other factors and processes, impacting carcinogenicity of EMPs). The properties of the particles that govern their ability to be deposited and then translocated elsewhere in the body include such interrelated characteristics as biopersistence, rigidity, dimension, density, and potential for disaggregation. These properties affect all parts of the journey from exposure to target tissue, and include transportation in the airways, deposition in the pulmonary system, translocation, and clearance. If an EMP must translocate from the lung to the parietal and peritoneal pleura in order to cause mesothelioma, which most accept as highly probable (33, 34), or be retained in the lung over a long period for lung cancer or lung fibrosis (asbestosis) to develop, then understanding the characteristics of particles deposited in the lung or capable of reaching the pleura and other organs would clarify the nature of mineral EMP carcinogens generally. In addition, dimensions and mineral composition govern the ability of EMPs to penetrate the membrane of target cells and potentially damage cell nuclei: another element of dose delivery that may be an important characteristic of an EMP carcinogens.

In our study, we developed a conception of the size distribution of inhaled elongate mineral particles as one of the major characteristics of the fiber deposition process. The concentrations of fibers in lungs play a very important role in understanding disease potential and provide unique information about the exposure. Actually, lung burden concentrations correlate to both cumulative exposure to various types of mineral fibers (11), and to the probability of mesothelioma (35). However, the size distribution of elongate particles in lungs provides unique information about the mechanisms of fiber deposition. The difference between clearance rate of fibers in lungs is driven by size and biopersistence of fibers (15).

We demonstrated that a special metric can be utilized to estimate a propensity of different size groups of fibers to be deposited in lung tissue. The coefficient DSR is defined as the ratio of the frequency of a specific size fraction in lungs vs. exposure. We used a groundbreaking publication by Pooley and Clark (13) as a basis for DSR calculation. For lung burden data, we utilized the information from mesothelioma cases and control populations, as published by Pooley and Clark. We used two sources of size distribution for particles in the exposure. The first approach was used by Pooley and Clark and included the airborne TEM measurements of fiber sizes in the exposure of workers from amosite and crocidolite mines in South Africa. As a second approach, we utilized information from the dimensional database, including different sources of amosite and crocidolite.

The determined DRS values provide a valuable insight about deposition mechanisms. We concluded that DSR values fluctuate in a wide range for amosite and crocidolite, starting with 0 (full deselection) and ending with 8.35 (significant preference). The highest preferential deposition for the size categories from Pooley and Clark is 8.35, observed for amosite for length category 8–10 μm, width<0.125 μm, in comparison to the dimensional database information.

We determined that DRS values can be approximated by a regression equation including log-transformed length (with positive coefficient) and width (with negative coefficient). It means that DRS increases with length and decreases with width. Pooley and Clark suggested that the “larger” particles had preference for lung deposition (Pooley, Clark meant “longer,” as we assume). We see that improved data demonstrates that specifically longer and narrower particles can be preferentially deposited.

We generated DRS estimations for each of the particles in the dimensional database, using the regression equation developed. Then we explore the difference in DRS for various subtypes of particles. In particular, we showed that non-asbestiform (cleavage fragments) amphiboles all have DRS<0.5, demonstrating the deselection process for cleavage fragments. This conclusion would not change if we used non-parametric estimation of DSR instead of the regression model. Miller et al. (36) correctly stated that of the fraction of cleavage fragments that may be respirable, their morphology allows for rapid clearance from the lungs by alveolar macrophages. However, cleavage fragments appear to be not deposited at all, having substantially larger aerodynamic diameter to penetrate deep lungs.

We also showed that DSR can be used to model mesothelioma potency of various mineral types of fibers, if supplemented by the biopersistence parameter for each mineral. Modeling of fiber potency by dimensional parameters, with chemical composition or biopersistence as additional variables, was shown to be effective (12, 37, 38). Our analysis demonstrates that DSR as a size-specific empirical parameter for lung deposition rate is closely related to dimensional-specific mesothelioma potency factors in amphiboles, chrysotile, single-chain silicates, and zeolites.

Roggli and Green examined a wide range of morphologic data for asbestos fibers recovered from the lungs of 91 human subjects (39). In that study, the majority of amosite and crocidolite fibers that persisted in the lungs after being inhaled have L > 10 μm and W < 1.0 μm. They concluded that the population of amosite and crocidolite fibers in lungs can be classified as asbestiform. Our analysis also shows amosite and crocidolite in lungs being asbestiform based on the developed dimensionality-based approach (18, 31).

We specifically noted, however, that there is a significant discrepancy between the US EPA MPPD estimations of pulmonary deposition for elongate particles and the observed lung deposition of particles. In our study, we determined that a correlation between the MPPD predictions of pulmonary deposition of elongate particles is statistically significantly negative, while it would be at least reasonable to expect a positive correlation, even if not a strong relationship. It is noteworthy that recently an experimental study by Rissler, et al. demonstrated imperfect fit of various deposition models with results of an experimental study on lung deposition of inhaled 2 μm particles (40). While the MPPD model provided the best approximation from the set of tested models, still experimental deposition rate was higher than all theoretical approximations by a factor of 2. While there is no well-developed literature on experimental testing for elongate mineral fiber bundles in comparison to deposition models, the results by Rissler et al., along with our study, suggest that there is a need to recommend improvements in the MPPD model developed and used by the US EPA in its application to elongate mineral particles.

It should be noted that the “deposition” process for particles in lungs, as interpreted in this paper, is a complex combination of sub-steps, such as lung penetration, retention, transformation, and clearance. In particular, some of thick particles will be going through disaggregation, with fraction of narrow particles increasing. Understanding of that should clarify our vision of “preferential” deposition of particles. The “preferential” deposition of particles in the lungs will be affected by the processes when new particles with new dimensional characteristics will be generated as a result of fragmentation.

The non-linear character of fiber clearance is also an important determinant of long-term fiber retention and overall carcinogenicity. Cumulative exposure is a powerful predictor of fiber deposition in lungs (11, 15, 35). Our study helps to address not only the concentrations of fibers in lungs as a function of cumulative exposure, but also size distribution of fibers, deposited in lungs, as a function of exposure size distribution.

Further studies are needed to explore concentrations of elongate particles in human lungs, especially with significant changes in exposure characteristics during the last decades. In any case, the re-exploration of original data from Pooley and Clark that we performed in this paper seems to be very important for our understanding of toxicokinetic processes in human lungs, and of the intrinsic differences between various types of fibers in producing malignant mesothelioma.

## 5 Limitations

There are uncertainties and limitations in our study. The information of lung burden of amosite and crocidolite in mesothelioma cases and controls, reported by Pooley and Clark, represents just a part of the extensive archive of human tissue samples collected by the Cardiff team. However, in our study we demonstrated how advanced methodological approaches may help to interpret even limited data on fibers in lung tissue, making it important material for toxicological analysis and risk assessment.

Table 7 demonstrates the comparison between central tendency (median) characteristics of amosite and crocidolite fibers in Pooley and Clark (13) (calculated based on the simulated datasets) with similar characteristics published by Warnock (41).

**Table 7:** Central tendency of dimensional parameters of fibers in lungs in comparison.

Source of information/mineral type	Length, μm (median)	Width, μm (median)	Aspect ratio (median)
Amosite			
Pooley and Clark	3.5	0.21	24
Warnock	3.9	0.17	23
Crocidolite			
Pooley and Clark	2.7	0.10	33
Warnock	3.8	0.09	40

We see overall consistency between dimensions of elongate particles detected in lungs by two sources.

The major limitation is that in each case we do not have a precise estimation of dimensional characteristics for specific exposure events. However, it appears that for amosite and crocidolite, airborne exposure can be characterized well based on a collection of various dimensional measurements. Further study would be needed to explore differences between occupational groups, to see if dimensions of fibers in lungs would be predictive of exposure characteristics.

Also, data from Pooley and Clark used a mixed set of mesothelioma and control cases. In future studies, it will be important to see commonalities and differences in lung concentrations of fibers based on pathology.

## 6 Conclusion

Based on available observations, length and width of elongate amphibole particles affect probability of their deposition in the lungs, that is reflected in the differences of size distribution of particles in the exposure and in human lungs.

Mathematical modeling show that probability of lung deposition for amphibole particles with various sizes increases with length and decreases with width.

Non-asbestiform particles (cleavage fragments) appear to be deselected by the deposition process, with prevailing habit of particles in lungs being asbestiform.

There is a significant difference between observed size distribution of particles in lungs and the distribution that would be predicted by US EPA MPPD model, that calls for further improvement of existing methods to estimate fiber transport and clearance.

## Data Availability

The original contributions presented in the study are included in the article/Supplementary material. Further inquiries can be directed to the corresponding author.

## Appendix A

**Table 1A:** Datasets from dimensional database used for the analysis.

Amosite	Crocidolite
Transvaal, SA (South Africa), NIST sample, Harper et al. (42) Transvaal, SA, simulated sample, Gibbs and Hwang (43) Transvaal, SA, NIST sample, Chatfield (personal communication) Pipe insulation, mixing, airborne, TEM, asbestiform, Dement and Harris (44) Pipe insulation, forming, airborne, TEM, asbestiform, Dement and Harris (44) Pipe insulation, finishing, airborne, TEM, asbestiform, Dement and Harris (44) Unleached sample, Cook et al. (33) Leached sample, Cook et al. (33)	Cape, SA (South Africa), crocidolite RTI sample (personal communication) South Africa, simulated, airborne sample, Gibbs and Hwang (43) South Africa, crocidolite, bulk sample, Shedd et al. (45) Wittenoom, Australia, crocidolite, Shedd et al. (45) NIST crocidolite sample, Chatfield (personal communication) Penarth, Wales, UICC crocidolite sample, Chatfield (personal communication) Kuruman Hill, SA, crocidolite sample, Chatfield (personal communication) Prieska mine, SA, elutriated sample, Chatfield (personal communication) Kuruman, SA, elutriated sample, Chatfield (personal communication) Pomfret, SA, elutriated sample, Chatfield (personal communication) Transvaal, SA, elutriated sample, Chatfield (personal communication) Wittenoom, Australia, Chatfield (personal communication) Bolivia, elutriated sample, Chatfield (personal communication) NIOSH, UICC crocidolite sample (personal communication) Unleached samples, Cook et al. (33) Leached samples, Cook et al. (33) Crocidolite sample, Wagner et al. (46) Crocidolite sample, airborne Gibbs and Hwang (43)
