# Supplementary material for: Size-dependent retention of elongate mineral particles in human lungs: modeling and implications for risk assessment
Source: Front Public Health. 2025 Sep 4;13:1646016. doi: 10.3389/fpubh.2025.1646016 (PMC12443763; doi:10.3389/fpubh.2025.1646016)
Supplement: Supplementary file 1 [file Table_1.docx]

Table S1. Size distribution of airborne fibers and fibers in human lungs based on Pooley and Clark (13)

| Length range (µm) | Fraction (%) in each length group | | Fraction (%) of fibers with specific diameter in a given length range | | | | | | | |
| --- | --- | --- | --- | --- | --- | --- | --- | --- | --- | --- |
|  |  |  | <0.125 µm | | 0.125-0.25 µm | | 0.25-0.375 µm | | >0.375 µm | |
|  | Crocidolite | Amosite | Crocidolite | Amosite | Crocidolite | Amosite | Crocidolite | Amosite | Crocidolite | Amosite |
| Lungs | | | | | | | | | | |
| <1 | 13.0 | 3.6 | 91.6 | 62.3 | 8.4 | 30.2 | - | 7.5 | - | - |
| 1-2 | 31.0 | 19.9 | 84.1 | 41.7 | 14.5 | 44.4 | 1.4 | 12.2 | - | 1.7 |
| 2-3 | 19.5 | 20.4 | 72.0 | 27.1 | 24.3 | 45.2 | 3.3 | 20.5 | 0.3 | 7.3 |
| 3-4 | 13.2 | 16.5 | 67.0 | 22.5 | 27.7 | 48.0 | 4.9 | 18.9 | 0.5 | 10.6 |
| 4-6 | 12.5 | 16.7 | 55.4 | 15.8 | 35.9 | 36.8 | 7.7 | 28.3 | 1.0 | 19.0 |
| 6-8 | 4.7 | 8.5 | 34.2 | 9.5 | 47.9 | 34.9 | 15.1 | 38.1 | 1.4 | 17.5 |
| 8-10 | 2.8 | 5.4 | 27.3 | 7.5 | 43.2 | 38.8 | 22.7 | 23.8 | 6.8 | 30.1 |
| >10 | 3.0 | 9.1 | 19.0 | 2.3 | 37.3 | 11.1 | 31.2 | 27.1 | 12.5 | 59.6 |
| Airborne | | | | | | | | | | |
| <1 | 24.0 | 12.3 | 78.8 | 55.8 | 20.8 | 39.0 | 0.7 | 5.2 | - | - |
| 1-2 | 37.8 | 26.2 | 57.4 | 21.3 | 37.4 | 46.3 | 4.8 | 22.0 | 0.4 | 10.3 |
| 2-3 | 15.0 | 18.8 | 35.2 | 14.4 | 49.5 | 30.5 | 8.8 | 22.9 | 6.6 | 32.2 |
| 3-4 | 8.6 | 10.9 | 28.8 | 8.8 | 53.8 | 35.3 | 7.7 | 25.0 | 9.6 | 30.9 |
| 4-6 | 8.0 | 13.7 | 34.7 | 8.1 | 46.9 | 30.2 | 12.2 | 25.6 | 6.1 | 36.0 |
| 6-8 | 3.3 | 5.7 | 25.0 | 5.9 | 45.0 | 35.3 | 10.0 | 8.8 | 20.0 | 50.0 |
| 8-10 | 2.0 | 4.8 | 25.0 | 6.7 | 33.3 | 30.0 | 33.3 | 20.0 | 8.3 | 43.3 |
| >10 | 1.3 | 7.9 | - | 10.2 | 37.5 | 18.3 | 37.5 | 14.3 | 25.0 | 57.2 |
